# Why Isn’t the Head Direction System Necessary for Direction? Lessons From the Lateral Mammillary Nuclei

**DOI:** 10.3389/fncir.2019.00060

**Published:** 2019-09-13

**Authors:** Christopher M. Dillingham, Seralynne D. Vann

**Affiliations:** School of Psychology, Cardiff University, Cardiff, United Kingdom

**Keywords:** spatial memory, rodent, head-direction cells, dosral tegmental nucleus of Gudden, anterodorsal thalamic nucleus

## Abstract

Complex spatial representations in the hippocampal formation and related cortical areas require input from the head direction system. However, a recurrent finding is that behavior apparently supported by these spatial representations does not appear to require input from generative head direction regions, i.e., lateral mammillary nuclei (LMN). Spatial tasks that tax direction discrimination should be particularly sensitive to the loss of head direction information, however, this has been repeatedly shown not to be the case. A further dissociation between electrophysiological properties of the head direction system and behavior comes in the form of geometric-based navigation which is impaired following lesions to the head direction system, yet head direction cells are not normally guided by geometric cues. We explore this apparent mismatch between behavioral and electrophysiological studies and highlight future experiments that are needed to generate models that encompass both neurophysiological and behavioral findings.

## Introduction

The ability to navigate through environments, and remember locations within those environments, is key to survival. Navigation requires a cognitive representation of both the environment and current position within the environment. Several positional correlates have been identified in the rodent brain, including hippocampal place cells (O’Keefe and Dostrovsky, 1971), which fire as a function of the animal’s position in two-dimensional space, while entorhinal grid cells constitute a boundary-defined representation of multiple, hexagonally arranged place fields (Hafting et al., 2005). Information relating to environmental features is represented by a number of classes of neurons including border cells, which fire in proximity to the boundaries of the environment (Hartley et al., 2000), and head direction cells (Taube et al., 1990), a class of cell that fires preferentially in reference to an animals’ directional heading in space.

Since their initial discovery in the postsubiculum (PoSub), head direction cells have been recorded in numerous other cortical and subcortical brain regions, including the retrosplenial (Chen et al., 1994; Cho and Sharp, 2001; Jacob et al., 2017), posterior parietal (PPC; Chen et al., 1994), medial entorhinal (MEC; Sargolini et al., 2006), and precentral cortices (Mehlman et al., 2019), the anterodorsal (ADN; Blair and Sharp, 1995; Taube, 1995), laterodorsal (Mizumori and Williams, 1993), anteroventral thalamic nuclei (Tsanov et al., 2011), nucleus reuniens (Jankowski et al., 2014), the dorsal striatum (Wiener, 1993; Mizumori et al., 2000, 2005; Ragozzino et al., 2001; Mehlman et al., 2019), the dorsal tegmental nucleus of Gudden (DTg; Sharp et al., 2001), and the lateral mammillary nuclei (LMN; Blair et al., 1998; Stackman and Taube, 1998). The traditional hierarchical model of the head direction system (for a more detailed recent review see Weiss and Derdikman, 2018) involves a vestibular/vestibulomotor-derived head direction signal which ascends to the LMN and is then updated through the integration of external sensory inputs, e.g., visual information from PoSub (Yoder et al., 2015), through to ADN, PoSub and MEC. At each ascending stage, cells receive updated, more complex input, which is reflected in the spatial information content of the neurons within the respective regions. For instance, a higher proportion of head direction cells exhibit a conjunctive head-direction/boundary signal in the PoSub than in ADN, which is thought to represent the integration of head direction with positional sensory input, e.g., whisking/optic flow, resulting from self-motion (Peyrache et al., 2017). An additional example of this hierarchical increase in complexity comes from bidirectional cells in the retrosplenial cortex (RSC; Jacob et al., 2017), which can represent two sensory modalities, i.e., olfaction and vision within a given environment (within compartment), or can represent a single sensory modality differently across contexts (between compartment).

While we know that the head direction system is necessary for accurate positional representation surprisingly little is understood about how this system contributes at a behavioral level. Manipulations of the LMN are particularly informative for studying the head direction system as these nuclei lie at the base of this highly interconnected hierarchy that integrates external cues, e.g., visual (Yoder et al., 2015), with those derived from self-motion (i.e., proprioceptive). Moreover, the high proportion of head direction cells in the LMN (Stackman and Taube, 1998; Taube and Bassett, 2003) allow for perturbation of the system while limiting confounding damage to additional pathways. In this review, we will consider what is currently known about the LMN’s contribution at an electrophysiological level, and at a behavioral level, and how this combined knowledge helps us understand the role of the head direction system in spatial cognition.

## How Does the Head Direction Signal Contribute to Spatial Signals Representing the Environment?

**Figure 1:**
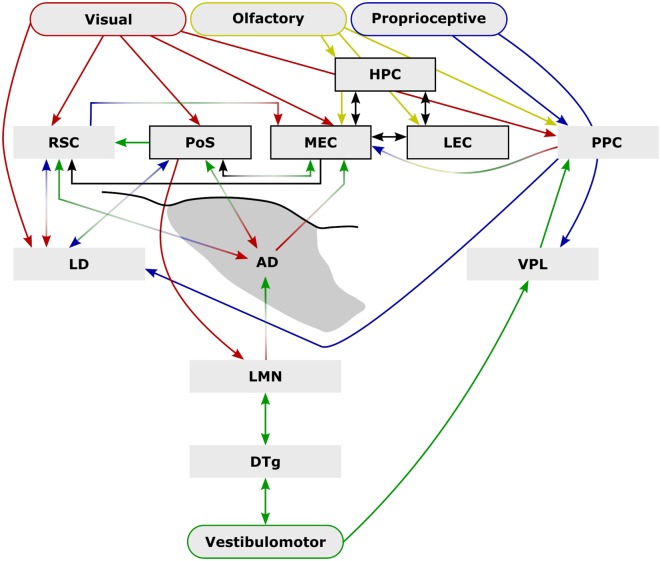
External sensory input is critical to spatial navigation. Ascending vestibulomotor (green) projections elicit directional tuning in many subcortical and cortical regions while descending visual (red), olfactory (yellow), and proprioceptive (blue) inputs are integrated to form complex physiological representations of space. Some of the extensive inter-communication between brain areas that encode space shown here demonstrate the capacity for compensation following disruption (e.g., lesion, or conflict between senses) while parallel pathways may provide additional compensatory mechanisms [e.g., vestibulomotor-ventral posterolateral thalamic nucleus (VPL)–posterior parietal cortex (PPC)] that may explain the mild behavioral impairment following lateral mammillary nucleus (LMN) or anterodorsal thalamic nucleus (ADN) lesions. Arrows indicate anatomical connections while their colors represent their possible sensory contributions. Structures and connections within the hippocampal formation that are not principally directional are shown black (arrows and boxes). Abbreviations: DTg, dorsal tegmental nucleus of Gudden; LD, laterodorsal thalamic nucleus; LEC, lateral entorhinal cortex; HPC, CA1–3 and dentate gyrus subfields of the hippocampal formation; MEC, medial entorhinal cortex; PoSub, postsubiculum; RSC, retrosplenial cortex.

Unlike hippocampal place cells whose spatial representations are context-dependent (e.g., Alme et al., 2014), the representation of head direction, border and grid cells maintain their intrinsic firing pattern across contexts (Hafting et al., 2005; Fyhn et al., 2007). Together these cells enable animals to encode features of an enclosed environment (Krupic et al., 2015) as well as positional information within that environment (Lever et al., 2009; Hinman et al., 2019). The head direction signal is seemingly important for both grid cell and place cell systems. Entorhinal grid cell periodicity is significantly disrupted following inactivation of the anterior thalamus, which includes ADN (Winter et al., 2015), although this could also reflect associated damage to the anteroventral and anteromedial thalamic nuclei e.g., through attenuation of MEC theta power (see Brandon et al., 2012). Grieves et al. (2016) showed that in multi-compartment environments, place cell repetition is more frequent if the compartments are in a parallel compared to radial configuration, suggesting direction information helps distinguish compartments. Consistent with this, LMN lesions increase place field repetition in radial compartments, likely reflecting the loss of directional information in these animals (Harland et al., 2017). While lesions of LMN do not degrade the spatial content of hippocampal place fields (Sharp and Koester, 2008), ADN lesions and, to a greater extent, PoSub lesions reduce the information content of place fields (Calton et al., 2003), albeit leaving the place fields intact. The increase in magnitude of impairment with each step along the ascending head direction pathway appears to co-occur with greater influence of descending visual inputs (V1-PoSub-RSC-ADN; Figure 1). Together these results suggest a hierarchical dependence of spatial systems that is borne out both by the sequence of anatomical inputs as well as the sequence of developmental emergence of spatial representations, i.e., directional tuning is fully developed earlier than place or grid cells (Langston et al., 2010; Wills et al., 2010). Grid cells appear to be the most complex of the spatial correlates so far identified, combining head direction, border and place signals. Consistent with this, inactivation of the hippocampus dramatically reduces the spatial information content of grid cells but interestingly leads to an increase in their directionality (Hafting et al., 2008; Bonnevie et al., 2013). While inactivation of the MEC does not degrade the spatial information content of hippocampal place fields it does induce place field remapping (Miao et al., 2015). However, large permanent lesions of the entorhinal cortex reduce place cell firing rate and spatial information content (Van Cauter et al., 2008) suggesting functional interdependence between grid cell and place cell networks.

## Do LMN Lesions Disrupt the Use of Directional Cues?

**Figure 2:**
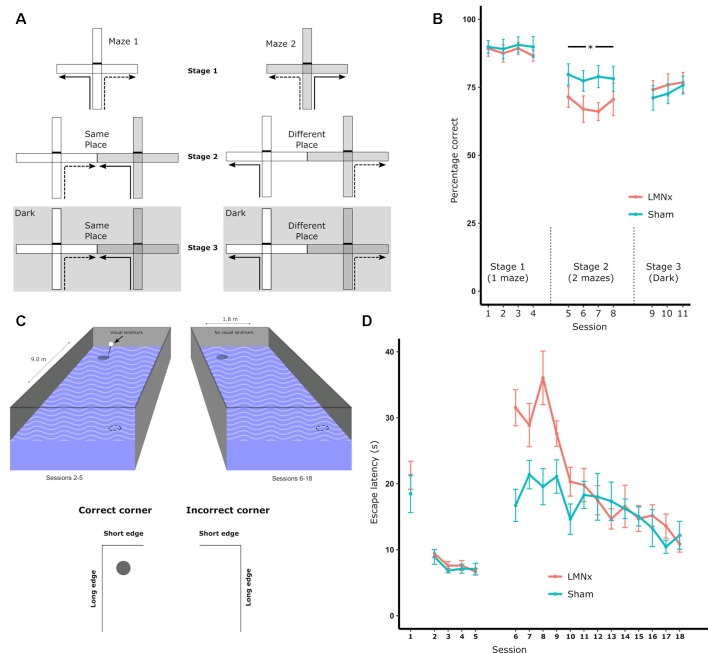
**(A)** Schematic of the T-maze alternation task: solid lines indicate the forced sample phase while dashed lines indicate the correct response in the choice phase. Arm access could be blocked by placing a barrier at the entrance of the arm (effectively turning the cross-maze into a T-maze configuration). Initial training on the task (Stage 1) permitted the use of multiple strategies supporting alternation, i.e., allocentric, intramaze, idiothetic, direction alternation (with reference to a known bearing). The task was then systematically modified in order to prevent the use of intramaze cues (Stage 2) or the use of intramaze and visual allocentric cues (Stage 3). These manipulations included using two mazes instead of one (Stage 2 and 3) or running in the dark, as illustrated with the dark gray hatched background (Stage 3); **(B)** percentage of correct choices for all three stages; **(C)** schematic of the geometric cue task. Rats were tested in a rectangular insert within a circular maze. A curtain was drawn around the maze throughout to encourage the use of intramaze cues for learning. The solid circles depict the submerged platform and the dotted circles indicate the geometrically identical platform location (on each trial there was only one platform). The visible landmark (black bar) was attached to the platform for Sessions 2–5 of the experiment; **(D)** the mean escape latencies for the two groups during training when they were required to find a platform beneath a landmark in a circular pool (Session 1), beneath a landmark in a rectangular pool (Session 2–5), and without a landmark in a rectangular pool (Session 5–18). The vertical lines depict the standard error of the mean. Abbreviations: LMNx, lateral mammillary nuclei lesion group; Sham, surgical control; *p < 0.05. Data are taken from Vann (2011).

Surprisingly, given the strong physiological influence of the head direction system on more spatially complex downstream systems, LMN lesions often have little to no effect on spatial tasks that would be presumed to involve a heading component. The reinforced T-maze task requires animals to alternate direction in a T-shaped maze in order to retrieve a reward (Figure 2A). In intact animals, head direction cells are landmark-locked to features of the maze (Dudchenko and Zinyuk, 2005) so it would be expected that disrupting the head direction system would disrupt T-maze performance. This is not the case as rats with neurotoxic LMN lesions show no impairment on standard T-maze alternation (Figure 2B; Vann, 2005, 2011). When performing the T-maze task, animals are able to use a number of different strategies, including relying on the use of allocentric, directional or intramaze cues. As a result, impairments in LMN lesion rats may be masked by animals using non-directional cues to perform the task. Modifying the task to restrict the cue-types that are available makes it possible to determine which cues animals are able to use. By carrying out sample and test runs in two separate adjacent mazes, animals are prevented from using intramaze or odor cues to perform the task. However, this manipulation also puts allocentric and directional cues into conflict for a subset of trials as alternating on the basis of direction requires animals to return to the same allocentric location (Figure 2A). Rats with neurotoxic lesions of the LMN show a mild impairment on this two-maze manipulation (Figure 2B; Vann, 2011). This impairment could reflect a greater reliance on intramaze cues or a greater sensitivity to the mismatch between direction and allocentric cues. This was examined by subsequently removing the mismatch between visual allocentric and direction cues by testing the animals in the dark (Figure 2A). This resulted in the lesion animals performing equivalently to the controls (Figure 2B). Together, these data suggest that animals with LMN lesions are less likely to use directional information when it is put in conflict with another spatial cue i.e., in this case visual allocentric information. However, when this conflict is removed, and direction cues become the most salient (Futter and Aggleton, 2006), animals with a disrupted head direction system are unimpaired (Vann, 2011).

In the T-maze task, animals have to choose between reward locations that are separated by 180°. This quite crude direction discrimination appears to be possible with an impaired head direction system. In contrast, the radial-arm maze task typically requires animals to discriminate between eight radially-oriented arms that are separated by 45°. This task would, therefore, be expected to require a more accurate directional representation. Additionally, LMN lesions increase place cell repetition in radially-oriented arms (Harland et al., 2017), which should also reduce animals’ ability to discriminate between the arms in a radial-arm maze. Nevertheless, lesions of the LMN failed to affect performance on this task (Vann, 2018). Furthermore, when manipulations were carried out to either reduce the reliance on intramaze cues or remove visual allocentric cues, LMN lesion rats were still unimpaired. Both of these manipulations would presumably increase reliance on internal direction systems, yet no lesion-induced impairment was found (Vann, 2018). It was not the case that the lesions in this experiment were ineffective, as the same animals were impaired on other spatial tasks and had reduced c-Fos expression in the RSC (Vann, 2011).

Watermaze tasks can be particularly useful for assessing spatial memory as they remove possible confounds of odor trails, the use of which can mask impairments in using allocentric spatial cues. Furthermore, watermaze tasks do not typically confine animals’ choices to specific “arms,” potentially enabling more subtle impairments in heading judgments to be detected. To date, two studies have looked at the contribution of the LMN to watermaze tasks carried out in a circular pool. The first study used a working memory procedure where animals were required to learn a new platform location within each session (Vann, 2005). LMN lesion animals showed an initial impairment on this task but with continued training their performance matched that of controls. This impairment is comparable to rats with discrete combined lesions of ADN/anteroventral thalamic nuclei where performance on a similar working memory task in the watermaze was only mildly impaired (van Groen et al., 2002). The second study looking at LMN contribution (Harland et al., 2015) tested animals on a reference memory task where the platform remained in the same spatial location across sessions. On this task, the LMN lesion rats performed equivalently to controls. However, when the platform was subsequently moved to the opposite spatial location (reversal) a lesion-induced deficit emerged. These findings suggest there is a dependence on the head direction system in situations that require rapid encoding of spatial information.

## The Effect of Head Direction System Lesions on Food-Carrying Tasks

Food-carrying tasks are typically run on open dry mazes with equally positioned entrances/exits around the edge, one of which contains a home refuge. The task requires animals to search for food on the maze and once animals have located the food, they carry it back to their refuge. As such, this task requires animals to keep track of their location within the environment, locate themselves with respect to their refuge, and then orient themselves to return to the refuge. It would, therefore, be expected that accurate performance on this task would depend on an intact head direction system. Consistent with this, lesions of a number of head direction areas have been found to impair animals’ accuracy of performance on this task (e.g., Frohardt et al., 2006; Clark and Taube, 2009; Dwyer et al., 2013; Peckford et al., 2014; Yoder et al., 2019).

To date, no study has successfully assessed the impact of selective LMN lesions on food-carrying tasks. However, comparisons with the findings from ADN lesions on this task could be particularly informative because LMN and ADN lesion effects appear to be quantitatively and qualitatively similar on other tasks (e.g., van Groen et al., 2002; Peckford et al., 2014). As with lesions to other head direction areas, ADN lesions impair performance on food-carrying tasks with animals more likely to return to a location adjacent to the refuge (Frohardt et al., 2006; Peckford et al., 2014). ADN lesions, therefore, result in a slightly less accurate angle of return indicating a role for the head direction system in fine-tuned direction discrimination. The assumption, therefore, is that LMN lesions would also result in a less accurate return trajectory on this task, consistent with previous LMN-lesion impairments observed on tasks that require rapid, flexible spatial learning.

A study which assessed the effects of large electrolytic DTg lesions on food-carrying tasks found impairments that were considerably greater than the impairments observed following ADN lesions (Frohardt et al., 2006). This could suggest that the DTg lesions reflect more than just the loss of the DTg-LMN-ADN head direction signal, i.e., the DTg could be influencing an additional pathway. The DTg is reciprocally connected with the supragenual nucleus (Biazoli et al., 2006; Clark et al., 2012a), and nucleus prepositus hypoglossi (Butler and Taube, 2015, 2017), both of which contain a significant proportion of angular head velocity cells and, in turn, are interconnected with the vestibular nuclei. However, lesions of the vestibular nuclei appear to increase animals’ reliance on the use of visual spatial cues to navigate while reducing their ability to use idiothetic cues (Stackman and Herbert, 2002), which seems very different from lesions of LMN where the use of visual spatial cues is impaired. Alternatively, given the location of the DTg, large electrolytic lesion may have substantial non-selective effects due to involvement of adjacent regions, e.g., dorsal raphe nuclei, and fibers of passage; this may explain DTg lesions appearing far more disruptive than lesions of LMN or ADN in some studies (Frohardt et al., 2006; Dwyer et al., 2013). Consistent with this interpretation, more discrete electrolytic DTg lesions impair rats’ ability to use visual-spatial cues while leaving intact their ability to use directional cues (Clark et al., 2013), a pattern of findings comparable to those found following LMN lesions (Vann, 2005, 2011).

## Is the Head Direction System a Geometric System?

Given the importance of geometrical cues for orientation and navigation, it is possible that the head direction system has a role in geometry-based navigation. To test this, rats with LMN lesions were required to learn the location of a platform in a rectangular watermaze on the basis of geometric features (Vann, 2011). The configuration of long and short walls results in the two diagonally-opposed corners of the rectangle being geometrically identical (Figure 2C). Consequently, there are two “correct” corners, which could contain a platform whereas the remaining two corners are incorrect (with no escape platform); rats had to learn to swim to the correct corners to escape. Rats with LMN lesions showed a striking impairment at the beginning of training, despite performing normally on pre-training procedures used to familiarize them with non-spatial aspects of the task (Figure 2D). Once again, however, the impairment was transient and, by the end of training, the lesion animals were performing at a level equivalent to the controls (Figure 2D; Vann, 2011). This geometric watermaze task appears particularly sensitive to disruption of the head direction system as lesions focused on the ADN also impair performance on this task whereas animals with lesions of the medial mammillary body system (Vann, 2013) or lesions of the fornix (Aggleton et al., 2009) appear unimpaired.

Stable, stimulus-locked sensory cues are critical to minimizing errors in cognitive representations of space, e.g., due to internally-generated drift in directional tuning in the absence of visual input (Taube et al., 1990; Goodridge et al., 1998). It would, therefore, be expected that salient geometric features of enclosed environments would be used preferentially by spatially-responsive cells and in subsequent navigational strategies (Gallistel, 1990). However, experimental evidence seems to suggest otherwise. For example, rotation of highly geometrically polarized enclosures is insufficient to drive rotations in the preferred firing direction of head direction cells throughout the LMN-ADN-PoSub-RSC axis (Knight et al., 2011). However, geometric cues do control head direction cell firing in animals that are disorientated (Knight et al., 2011; Clark et al., 2012b) suggesting that while geometric cues can control head direction cell activity, they are not used preferentially. One explanation is that while geometric features are stable, they are not necessarily visually salient, and their recognition is reliant on the detection of how light is differentially reflected off surfaces. In low or dim light the contrast in luminance would be lower such that updating positional information from these features would be more reliant on sensory inputs that require proximity to the feature, e.g., somatosensory/haptic flow (particularly so given the relatively poor resolution of vision in rodents). When behavioral studies specifically require the use of geometric cues, these cues appear less salient to animals with an impaired head direction system, i.e., lesion animals are less readily able to use these cues to navigate. However, this initial impairment can be overcome with repeated training suggesting the head direction contribution may be important for directing initial attention to the geometric cues (Vann, 2011), particularly so in aversive conditions when rapid learning is required.

## What Compensatory Mechanisms Support Function Following LMN Lesions?

As described, behavioral deficits observed following LMN lesions tend to be very mild and/or transient, so it is likely that compensatory mechanisms are able to support function when this directional pathway is lost. These mechanisms include behavioral compensation whereby animals adopt different behavioral strategies to perform the tasks or functional compensation where different brain regions are able to support similar functions to mitigate the effects of the lesion. For instance, while Yoder et al. (2019) found that PoSub lesions in naïve animals caused impairments in path integration (food-carrying task), Bett et al. (2012) found no impairment in PoSub lesion animals that had been pre-trained prior to surgery. Pre-training likely acts as a compensatory mechanism by enabling animals to use previously learned strategies (Yoder et al., 2019) and/or form neurally-distributed representations of the environment which could better support navigation when directional information is limited.

First, we will consider behavioral compensation. Animals can often use a number of different cues and strategies to perform spatial tasks. In particular, tasks where animals have limited options, such as T-maze alternation, can often be supported by more habitual striatal-based behavior (Valerio et al., 2010; Gibson et al., 2013). The idea that animals use different strategies when head direction information is limited is additionally supported by work using an inverted navigation approach. The head direction signal is severely degraded when rats navigate upside-down (Taube et al., 2004; Calton and Taube, 2005; Gibson et al., 2013) making it possible to assess the head direction system’s contribution to navigation without using surgical intervention. Rats tasked with navigating towards a goal location (i.e., an escape hole), while inverted, were impaired when there were four possible release locations but performance was intact when only two release locations were used as long as salient visual landmarks were available (Valerio et al., 2010). It appears that the head direction signal is needed when flexible navigation is required but the use of a habit-like strategy could support performance in some conditions. This is a similar to findings from LMN-lesion studies. Impairments are often most pronounced when animals have to adapt behavior or respond flexibly, as is the case during both the reversal and working memory tasks in the watermaze. Both the radial-arm maze task and water maze task are unlikely to be supported by habit-like strategies from the outset but it appears that when learning is slow and incremental, again the head direction system is not so critical. Together these highlight the importance of probing behavioral impairments to determine exactly how animals are performing the task and what cues are being used. A consistent finding, however, is that rapid, flexible learning appears most sensitive to lesions within the head direction systems.

An additional explanation for the mild lesion effects following LMN lesions is that there is redundancy within the head direction system. Initially there just appeared to be a single head direction pathway, however, now it is apparent that there is a distributed head direction system across numerous brain regions. At present, it is not clear how these additional areas relate to the LMN-based system. For example, lesions of the LMN result in a small reduction in RSC activity, as measured by the immediate early gene c-Fos (Vann, 2018), but it is not known whether the retrosplenial head direction signal is dependent on indirect inputs from LMN. From current anatomical knowledge, it would not be expected that the head direction signal in the anteroventral nucleus, nucleus reuniens, laterodorsal thalamus or the striatum would depend on direct inputs from the LMN (Dillingham et al., 2015). So it is possible that even with the loss of LMN there is sufficient directional information to support task performance. Consistent with this idea, there is anatomical evidence for parallel streams of ascending vestibular projections that may also provide functional compensation (Figure 1). One such possibility involves projections from the vestibular nuclei which ascend to the ventral posterolateral thalamus (VPL), contributing to a proprioceptive representation in the PPC (McNaughton et al., 1989; Mimica et al., 2018). Vestibulomotor, i.e., idiothetic, information is sufficient to generate a head direction signal (Goodridge and Taube, 1997; Calton and Taube, 2009; Clark et al., 2010; Clark and Taube, 2011; Yoder et al., 2015; Mehlman et al., 2019) and it is possible that parallel ascending vestibular-VPL-PPC projections subserve a parallel directional pathway. The PPC, in turn, projects strongly to the parahippocampal region, including direct projections to the MEC as well as reciprocal connections with the RSC. Lesions of the DTg (Bassett et al., 2007), LMN (Blair et al., 1999), or ADN abolish the head direction signal in the PoSub (Goodridge and Taube, 1997) and the MEC (Winter et al., 2015). It would, therefore, be expected that if the vestibular-VPL-PPC axis does provide a parallel source of heading information, lesions within the LMN-ADN pathway would spare the PPC head direction signal, however, to our knowledge, this has not yet been tested.

Unlike medial mammillary projections to the anterior thalamic nuclei, projections from the LMN to the ADN are bilateral, which provides a degree of anatomical compensation. Blair et al. (1999) compared the effects of unilateral and bilateral LMN lesions on head direction firing in ADN and found that while unilateral lesions induced some impairments in ADN directional tuning immediately following the lesion, they were transient, and recovery of function was evident within a few days. Consistent with other studies (Blair et al., 1998; Bassett et al., 2007), however, bilateral LMN lesions abolished directional firing permanently. Head direction cell firing in LMN that results from head movements towards the hemisphere in which the cell is located, i.e., ipsiversive turns, result in narrower tuning curves than contraversive turns (Blair et al., 1998). Following the transient effects of unilateral LMN lesions, head direction tuning curves in ADN were found to be narrower in response to head turns in the direction of the intact hemisphere, suggesting that compensation to the lesion was, at least in part, due to an increase in the influence of intact contralateral projections (Blair et al., 1999). In addition to the loss of ADN head direction cell tuning following bilateral LMN lesions, there was an emergence of increased theta band-entrained firing activity along with velocity-dependent firing. These changes again may point towards a level of plasticity through either an increased responsiveness to non-directional inputs or through an increased reliance on non-vestibular directional sense, e.g., *via* somatosensory-laterodorsal thalamic nucleus connections (Bezdudnaya and Keller, 2008).

## Discussion

At a neurophysiological level, the generative network of the head direction system (Figure 1) is critical for complex spatial processing in downstream structures. However, this neurophysiological importance is not mirrored by the scale of the behavioral impairments observed following lesions to this network, particularly with respect to the LMN. It is perhaps unsurprising that given the evolutionary importance of effective navigational strategies, there is considerable scope for compensation, e.g., through dependence on multiple external cues (Figure 1). Compared to other neural correlates of space (e.g., place cells, grid cells, object cell, boundary-vector cells), subcortical head direction cells are relatively rudimentary in the information they encode, i.e., a representation of the position of the head in a single plane (yaw) with respect to a salient visual landmark, without providing other metrics, e.g., distance from landmarks (but see Peyrache et al., 2017). In that sense, they may be seen as both the product of combined representations, e.g., vestibular and motor (i.e., angular velocity), as well as the building blocks to more informative representations of space, i.e., attractor network models. An effective cognitive spatial representation must also be rapidly updated both with respect to changes that result from self-motion as well as in response to external changes and in that sense, subcortical head direction cells have their limitations, e.g., in the absence of visual cues, drift in directional tuning represents a less stable substrate for path integration than the relative stability of the hippocampal place signal (Save et al., 2000).

We have known of the existence of head direction cells for 30 years (Taube et al., 1990) yet our understanding of their behavioral contributions is surprisingly lacking. There are remarkably few studies that have studied behavioral contributions of these structures. Lesions of these regions are technically difficult as they are typically small structures that are adjacent to other key spatial memory regions. When lesions encroach into adjacent regions the results can be very difficult to interpret. A further issue is interpreting lesions of head direction regions in terms of head direction. Head direction cells typically make up the minority of cells within these structures (proportions of head direction cells in regions within the DTg-LMN-ADN-PoSub axis range from 12.5% to 60%; Taube and Bassett, 2003); it is likely that non-head direction cells also contribute to these tasks, making the behavioral contribution of the head direction system less than assumed. In light of compensatory mechanisms that might mask behavioral effects of LMN/DTg lesions, chronic lesion paradigms, which are typically employed within the head direction-system literature, may not be sufficiently sensitive. Approaches that use reversible inactivation with high temporal precision, e.g., optogenetics, may be more informative in this sense (e.g., Butler et al., 2017). Combining such techniques with approaches that permit the targeting of distinct neuronal populations that share common anatomical connectivity and/or neurochemical properties could enable temporary inactivation of head direction cells while leaving other cells intact. It is essential to combine these techniques with well-designed behavioral studies if we are to solve the puzzle of how the head direction system contributes to spatial navigation.

## Author Contributions

CD and SV both conceived and wrote the article.

## Conflict of Interest Statement

The authors declare that the research was conducted in the absence of any commercial or financial relationships that could be construed as a potential conflict of interest.

## References

[B1] Aggleton J. P. Poirier G. L. Aggleton H. S. Vann S. D. Pearce J. M. (2009). Lesions of the fornix and anterior thalamic nuclei dissociate different aspects of hippocampal-dependent spatial learning: implications for the neural basis of scene learning. Behav. Neurosci. 123, 504–519. 10.1037/a0015404 19485556

[B2] Alme C. B. Miao C. Jezek K. Treves A. Moser E. I. Moser M. B. (2014). Place cells in the hippocampus: eleven maps for eleven rooms. Proc. Natl. Acad. Sci. U S A 111, 18428–18435. 10.1073/pnas.1421056111 25489089 PMC4284589

[B3] Bassett J. P. Tullman M. L. Taube J. S. (2007). Lesions of the tegmentomammillary circuit in the head direction system disrupt the head direction signal in the anterior thalamus. J. Neurosci. 27, 7564–7577. 10.1523/JNEUROSCI.0268-07.2007 17626218 PMC6672597

[B600] Bett D. Wood E. R. Dudchenko P. A. (2012). The postsubiculum is necessary for spatial alternation but not for homing by path integration. Behav. Neurosci. 126, 237–248. 10.1037/a0027163 22352792

[B4] Bezdudnaya T. Keller A. (2008). Laterodorsal nucleus of the thalamus: a processor of somatosensory inputs. J. Comp. Neurol. 507, 1979–1989. 10.1002/cne.21664 18273888 PMC2800129

[B5] Biazoli C. E. Jr. Goto M. Campos A. M. Canteras N. S. (2006). The supragenual nucleus: a putative relay station for ascending vestibular signs to head direction cells. Brain Res. 1094, 138–148. 10.1016/j.brainres.2006.03.101 16684515

[B6] Blair H. T. Cho J. W. Sharp P. E. (1998). Role of the lateral mammillary nucleus in the rat head direction circuit: a combined single unit recording and lesion study. Neuron 21, 1387–1397. 10.1016/s0896-6273(00)80657-1 9883731

[B7] Blair H. T. Cho J. W. Sharp P. E. (1999). The anterior thalamic head-direction signal is abolished by bilateral but not unilateral lesions of the lateral mammillary nucleus. J. Neurosci. 19, 6673–6683. 10.1523/JNEUROSCI.19-15-06673.1999 10414996 PMC6782818

[B1000] Blair H. T. Sharp P. E. (1995). Anticipatory head direction signals in anterior thalamus: evidence for a thalamocortical circuit that integrates angular head motion to compute head direction. J. Neurosci. 15, 6260–6270. 10.1523/JNEUROSCI.15-09-06260.1995 7666208 PMC6577663

[B8] Bonnevie T. Dunn B. Fyhn M. Hafting T. Derdikman D. Kubie J. L.. (2013). Grid cells require excitatory drive from the hippocampus. Nat. Neurosci. 16, 309–317. 10.1038/nn.3311 23334581

[B9] Brandon M. P. Bogaard A. R. Andrews C. M. Hasselmo M. E. (2012). Head direction cells in the postsubiculum do not show replay of prior waking sequences during sleep. Hippocampus 22, 604–618. 10.1002/hipo.20924 21509854 PMC3288437

[B10] Butler W. N. Taube J. S. (2015). The nucleus prepositus hypoglossi contributes to head direction cell stability in rats. J. Neurosci. 35, 2547–2558. 10.1523/JNEUROSCI.3254-14.2015 25673848 PMC4323533

[B12] Butler W. N. Smith K. S. van der Meer M. A. A. Taube J. S. (2017). The head-direction signal plays a functional role as a neural compass during navigation. Curr. Biol. 27, 1259–1267. 10.1016/j.cub.2017.03.033 28416119 PMC5425164

[B11] Butler W. N. Taube J. S. (2017). Oscillatory synchrony between head direction cells recorded bilaterally in the anterodorsal thalamic nuclei. J. Neurophysiol. 117, 1847–1852. 10.1152/jn.00881.2016 28250151 PMC5411469

[B14] Calton J. L. Stackman R. W. Goodridge J. P. Archey W. B. Dudchenko P. A. Taube J. S. (2003). Hippocampal place cell instability after lesions of the head direction cell network. J. Neurosci. 23, 9719–9731. 10.1523/JNEUROSCI.23-30-09719.2003 14585999 PMC6740880

[B1300] Calton J. L. Taube J. S. (2005). Degradation of head direction cell activity during inverted locomotion. J. Neurosci. 25, 2420–2428. 10.1523/JNEUROSCI.3511-04.2005 15745969 PMC6726092

[B13] Calton J. L. Taube J. S. (2009). Where am I and how will I get there from here? A role for posterior parietal cortex in the integration of spatial information and route planning. Neurobiol. Learn. Mem. 91, 186–196. 10.1016/j.nlm.2008.09.015 18929674 PMC2666283

[B15] Chen L. L. Lin L. H. Green E. J. Barnes C. A. McNaughton B. L. (1994). Head-direction cells in the rat posterior cortex. I. Anatomical distribution and behavioral modulation. Exp. Brain Res. 101, 8–23. 10.1007/bf00243212 7843305

[B16] Cho J. Sharp P. E. (2001). Head direction, place, and movement correlates for cells in the rat retrosplenial cortex. Behav. Neurosci. 115, 3–25. 10.1037/0735-7044.115.1.3 11256450

[B19] Clark B. J. Bassett J. P. Wang S. S. Taube J. S. (2010). Impaired head direction cell representation in the anterodorsal thalamus after lesions of the retrosplenial cortex. J. Neurosci. 30, 5289–5302. 10.1523/JNEUROSCI.3380-09.2010 20392951 PMC2861549

[B20] Clark B. J. Brown J. E. Taube J. S. (2012a). Head direction cell activity in the anterodorsal thalamus requires intact supragenual nuclei. J. Neurophysiol. 108, 2767–2784. 10.1152/jn.00295.2012 22875899 PMC3545120

[B21] Clark B. J. Harris M. J. Taube J. S. (2012b). Control of anterodorsal thalamic head direction cells by environmental boundaries: comparison with conflicting distal landmarks. Hippocampus 22, 172–187. 10.1002/hipo.20880 21080407

[B22] Clark B. J. Rice J. P. Akers K. G. Candelaria-Cook F. T. Taube J. S. Hamilton D. A. (2013). Lesions of the dorsal tegmental nuclei disrupt control of navigation by distal landmarks in cued, directional, and place variants of the Morris water task. Behav. Neurosci. 127, 566–581. 10.1037/a0033087 23731069 PMC3997071

[B17] Clark B. J. Taube J. S. (2009). Deficits in landmark navigation and path integration after lesions of the interpeduncular nucleus. Behav. Neurosci. 123, 490–503. 10.1037/a0015477 19485555 PMC2698129

[B18] Clark B. J. Taube J. S. (2011). Intact landmark control and angular path integration by head direction cells in the anterodorsal thalamus after lesions of the medial entorhinal cortex. Hippocampus 21, 767–782. 10.1002/hipo.20874 21049489 PMC5723439

[B23] Dillingham C. M. Frizzati A. Nelson A. J. Vann S. D. (2015). How do mammillary body inputs contribute to anterior thalamic function? Neurosci. Biobehav. Rev. 54, 108–119. 10.1016/j.neubiorev.2014.07.025 25107491 PMC4462591

[B170] Dudchenko P. A. Zinyuk L. E. (2005). The formation of cognitive maps of adjacent environments: evidence from the head direction cell system. Behav. Neurosci. 119, 1511–1523. 10.1037/0735-7044.119.6.1511 16420155

[B24] Dwyer J. A. Ingram M. L. Snow A. C. Thorpe C. M. Martin G. M. Skinner D. M. (2013). The effects of bilateral lesions to the dorsal tegmental nucleus on spatial learning in rats. Behav. Neurosci. 127, 867–877. 10.1037/a0034931 24341711

[B25] Frohardt R. J. Bassett J. P. Taube J. S. (2006). Path integration and lesions within the head direction cell circuit: comparison between the roles of the anterodorsal thalamus and dorsal tegmental nucleus. Behav. Neurosci. 120, 135–149. 10.1037/0735-7044.120.1.135 16492124

[B26] Futter J. E. Aggleton J. P. (2006). How rats perform spatial working memory tasks: limitations in the use of egocentric and idiothetic working memory. Q. J. Exp. Psychol. 59, 77–99. 10.1080/02724990544000068 16556560

[B27] Fyhn M. Hafting T. Treves A. Moser M. B. Moser E. I. (2007). Hippocampal remapping and grid realignment in entorhinal cortex. Nature 446, 190–194. 10.1038/nature05601 17322902

[B28] Gallistel C. R. (1990). Representations in animal cognition: an introduction. Cognition 37, 1–22. 10.1016/0010-0277(90)90016-d 2269003

[B29] Gibson B. Butler W. N. Taube J. S. (2013). The head-direction signal is critical for navigation requiring a cognitive map but not for learning a spatial habit. Curr. Biol. 23, 1536–1540. 10.1016/j.cub.2013.06.030 23891111 PMC4106916

[B30] Goodridge J. P. Taube J. S. (1997). Interaction between the postsubiculum and anterior thalamus in the generation of head direction cell activity. J. Neurosci. 17, 9315–9330. 10.1523/JNEUROSCI.17-23-09315.1997 9364077 PMC6573595

[B31] Goodridge J. P. Dudchenko P. A. Worboys K. A. Golob E. J. Taube J. S. (1998). Cue control and head direction cells. Behav. Neurosci. 112, 749–761. 10.1037/0735-7044.112.4.749 9733184

[B3200] Grieves R. M. Jenkins B. W. Harland B. C. Wood E. R. Dudchenko P. A. (2016). Place field repetition and spatial learning in a multicompartment environment. Hippocampus 26, 118–134. 10.1002/hipo.22496 26190393 PMC4745022

[B32] Hafting T. Fyhn M. Bonnevie T. Moser M. B. Moser E. I. (2008). Hippocampus-independent phase precession in entorhinal grid cells. Nature 453, 1248–1252. 10.1038/nature06957 18480753

[B33] Hafting T. Fyhn M. Molden S. Moser M. B. Moser E. I. (2005). Microstructure of a spatial map in the entorhinal cortex. Nature 436, 801–806. 10.1038/nature03721 15965463

[B34] Harland B. Grieves R. M. Bett D. Stentiford R. Wood E. R. Dudchenko P. A. (2017). Lesions of the head direction cell system increase hippocampal place field repetition. Curr. Biol. 27, 2706.e2–2712.e2. 10.1016/j.cub.2017.07.071 28867207 PMC5607353

[B35] Harland B. Wood E. R. Dudchenko P. A. (2015). The head direction cell system and behavior: the effects of lesions to the lateral mammillary bodies on spatial memory in a novel landmark task and in the water maze. Behav. Neurosci. 129, 709–719. 10.1037/bne0000106 26501176 PMC4655868

[B36] Hartley T. Burgess N. Lever C. Cacucci F. O’Keefe J. (2000). Modeling place fields in terms of the cortical inputs to the hippocampus. Hippocampus 10, 369–379. 10.1002/1098-1063(2000)10:4<369::aid-hipo3>3.0.co;2-0 10985276

[B37] Hinman J. R. Chapman G. W. Hasselmo M. E. (2019). Neuronal representation of environmental boundaries in egocentric coordinates. Nat. Commun. 10:2772. 10.1038/s41467-019-10722-y 31235693 PMC6591168

[B38] Jacob P. Y. Casali G. Spieser L. Page H. Overington D. Jeffery K. (2017). An independent, landmark-dominated head-direction signal in dysgranular retrosplenial cortex. Nat. Neurosci. 20, 173–175. 10.1038/nn.4465 27991898 PMC5274535

[B39] Jankowski M. M. Islam M. N. Wright N. F. Vann S. D. Erichsen J. T. Aggleton J. P.. (2014). Nucleus reuniens of the thalamus contains head direction cells. Elife 3:e03075. 10.7554/elife.03075 25024427 PMC4115655

[B40] Knight R. Hayman R. Lin Ginzberg L. Jeffery K. (2011). Geometric cues influence head direction cells only weakly in nondisoriented rats. J. Neurosci. 31, 15681–15692. 10.1523/JNEUROSCI.2257-11.2011 22049411 PMC3242014

[B41] Krupic J. Bauza M. Burton S. Barry C. O’Keefe J. (2015). Grid cell symmetry is shaped by environmental geometry. Nature 518, 232–235. 10.1038/nature14153 25673417 PMC4576734

[B42] Langston R. F. Ainge J. A. Couey J. J. Canto C. B. Bjerknes T. L. Witter M. P.. (2010). Development of the spatial representation system in the rat. Science 328, 1576–1580. 10.1126/science.1188210 20558721

[B43] Lever C. Burton S. Jeewajee A. O’Keefe J. Burgess N. (2009). Boundary vector cells in the subiculum of the hippocampal formation. J. Neurosci. 29, 9771–9777. 10.1523/JNEUROSCI.1319-09.2009 19657030 PMC2736390

[B44] McNaughton B. L. Leonard B. Chen L. (1989). Cortical-hippocampal interactions and cognitive mapping: a hypothesis based on reintegration of the parietal and inferotemporal pathways for visual processing. Psychobiology 17, 230–235. 10.1007/bf03337774

[B45] Mehlman M. L. Winter S. S. Valerio S. Taube J. S. (2019). Functional and anatomical relationships between the medial precentral cortex, dorsal striatum and head direction cell circuitry. I. Recording studies. J. Neurophysiol. 121, 350–370. 10.1152/jn.00143.2018 30427767 PMC6397396

[B46] Miao C. Cao Q. Ito H. T. Yamahachi H. Witter M. P. Moser M. B.. (2015). Hippocampal remapping after partial inactivation of the medial entorhinal cortex. Neuron 88, 590–603. 10.1016/j.neuron.2015.09.051 26539894

[B47] Mimica B. Dunn B. A. Tombaz T. Bojja V. Whitlock J. R. (2018). Efficient cortical coding of 3D posture in freely behaving rats. Science 362, 584–589. 10.1126/science.aau2013 30385578

[B48] Mizumori S. J. Williams J. D. (1993). Directionally selective mnemonic properties of neurons in the lateral dorsal nucleus of the thalamus of rats. J. Neurosci. 13, 4015–4028. 10.1523/JNEUROSCI.13-09-04015.1993 8366357 PMC6576470

[B49] Mizumori S. J. Canfield J. G. Yeshenko O. (2005). Parallel and interrelated neural systems underlying adaptive navigation. Integr. Comp. Biol. 45, 547–554. 10.1093/icb/45.3.547 21676800

[B50] Mizumori S. J. Cooper B. G. Leutgeb S. Pratt W. E. (2000). A neural systems analysis of adaptive navigation. Mol. Neurobiol. 21, 57–82. 10.1385/mn:21:1-2:057 11327150

[B51] O’Keefe J. Dostrovsky J. (1971). The hippocampus as a spatial map. Preliminary evidence from unit activity in the freely-moving rat. Brain Res. 34, 171–175. 10.1016/0006-8993(71)90358-1 5124915

[B52] Peckford G. Dwyer J. A. Snow A. C. Thorpe C. M. Martin G. M. Skinner D. M. (2014). The effects of lesions to the postsubiculum or the anterior dorsal nucleus of the thalamus on spatial learning in rats. Hippocampus 128, 654–665. 10.1037/bne0000019 25420126

[B53] Peyrache A. Schieferstein N. Buzsaki G. (2017). Transformation of the head-direction signal into a spatial code. Nat. Commun. 8:1752. 10.1038/s41467-017-01908-3 29170377 PMC5700966

[B54] Ragozzino K. E. Leutgeb S. Mizumori S. J. (2001). Dorsal striatal head direction and hippocampal place representations during spatial navigation. Exp. Brain Res. 139, 372–376. 10.1007/s002210100795 11545476

[B55] Sargolini F. Fyhn M. Hafting T. McNaughton B. L. Witter M. P. Moser M. B.. (2006). Conjunctive representation of position, direction and velocity in entorhinal cortex. Science 312, 758–762. 10.1126/science.1125572 16675704

[B5700] Save E. Nerad L. Poucet B. (2000). Contribution of multiple sensory information to place field stability in hippocampal place cells. Hippocampus 10, 64–76. 10.1002/(SICI)1098-1063(2000)10:1<64::AID-HIPO7>3.0.CO;2-Y 10706218

[B56] Sharp P. E. Koester K. (2008). Lesions of the mammillary body region severely disrupt the cortical head direction, but not place cell signal. Hippocampus 18, 766–784. 10.1002/hipo.20436 18446828

[B57] Sharp P. E. Tinkelman A. Cho J. (2001). Angular velocity and head direction signals recorded from the dorsal tegmental nucleus of gudden in the rat: implications for path integration in the head direction cell circuit. Behav. Neurosci. 115, 571–588. 10.1037/0735-7044.115.3.571 11439447

[B58] Stackman R. W. Herbert A. M. (2002). Rats with lesions of the vestibular system require a visual landmark for spatial navigation. Behav. Brain Res. 128, 27–40. 10.1016/s0166-4328(01)00270-4 11755687

[B59] Stackman R. W. Taube J. S. (1998). Firing properties of rat lateral mammillary single units: head direction, head pitch and angular head velocity. J. Neurosci. 18, 9020–9037. 10.1523/JNEUROSCI.18-21-09020.1998 9787007 PMC1550347

[B6600] Taube J. S. (1995). Head direction cells recorded in the anterior thalamic nuclei of freely moving rats. J. Neurosci. 15, 70–86. 10.1523/JNEUROSCI.15-01-00070.1995 7823153 PMC6578288

[B60] Taube J. S. Bassett J. P. (2003). Persistent neural activity in head direction cells. Cereb. Cortex 13, 1162–1172. 10.1093/cercor/bhg102 14576208

[B61] Taube J. S. Muller R. U. Ranck J. B. Jr. (1990). Head-direction cells recorded from the postsubiculum in freely moving rats. II. Effects of environmental manipulations. J. Neurosci. 10, 436–447. 10.1523/JNEUROSCI.10-02-00436.1990 2303852 PMC6570161

[B6400] Taube J. S. Stackman R. W. Calton J. L. Oman C. M. (2004). Rat head direction cell responses in zero-gravity parabolic flight. J. Neurophysiol. 92, 2887–2997. 10.1152/jn.00887.2003 15212426

[B62] Tsanov M. Chah E. Vann S. D. Reilly R. B. Erichsen J. T. Aggleton J. P.. (2011). Theta-modulated head direction cells in the rat anterior thalamus. J. Neurosci. 31, 9489–9502. 10.1523/JNEUROSCI.0353-11.2011 21715614 PMC3855197

[B63] Valerio S. Clark B. J. Chan J. H. Frost C. P. Harris M. J. Taube J. S. (2010). Directional learning, but no spatial mapping by rats performing a navigational task in an inverted orientation. Neurobiol. Learn. Mem. 93, 495–505. 10.1016/j.nlm.2010.01.007 20109566 PMC2862784

[B64] Van Cauter T. Poucet B. Save E. (2008). Unstable CA1 place cell representation in rats with entorhinal cortex lesions. Eur. J. Neurosci. 27, 1933–1946. 10.1111/j.1460-9568.2008.06158.x 18412614

[B65] van Groen T. Kadish I. Michael Wyss J. (2002). Role of the anterodorsal and anteroventral nuclei of the thalamus in spatial memory in the rat. Behav. Brain Res. 132, 19–28. 10.1016/s0166-4328(01)00390-4 11853854

[B66] Vann S. D. (2005). Transient spatial deficit associated with bilateral lesions of the lateral mammillary nuclei. Eur. J. Neurosci. 21, 820–824. 10.1111/j.1460-9568.2005.03896.x 15733102

[B67] Vann S. D. (2011). A role for the head-direction system in geometric learning. Behav. Brain Res. 224, 201–206. 10.1016/j.bbr.2011.05.033 21672560

[B68] Vann S. D. (2013). Dismantling the Papez circuit for memory in rats. Elife 2:e00736. 10.7554/elife.00736 23805381 PMC3691571

[B69] Vann S. D. (2018). Lesions within the head direction system reduce retrosplenial c-fos expression but do not impair performance on a radial-arm maze task. Behav. Brain Res 338, 153–158. 10.1016/j.bbr.2017.10.026 29079513 PMC5701769

[B70] Weiss S. Derdikman D. (2018). Role of the head-direction signal in spatial tasks: when and how does it guide behavior? J. Neurophysiol. 120, 78–87. 10.1152/jn.00560.2017 29537921

[B71] Wiener S. I. (1993). Spatial and behavioral correlates of striatal neurons in rats performing a self-initiated navigation task. J. Neurosci. 13, 3802–3817. 10.1523/JNEUROSCI.13-09-03802.1993 8366346 PMC6576451

[B72] Wills T. J. Cacucci F. Burgess N. O’Keefe J. (2010). Development of the hippocampal cognitive map in preweanling rats. Science 328, 1573–1576. 10.1126/science.1188224 20558720 PMC3543985

[B73] Winter S. S. Clark B. J. Taube J. S. (2015). Spatial navigation. Disruption of the head direction cell network impairs the parahippocampal grid cell signal. Science 347, 870–874. 10.1126/science.1259591 25700518 PMC4476794

[B74] Yoder R. M. Peck J. R. Taube J. S. (2015). Visual landmark information gains control of the head direction signal at the lateral mammillary nuclei. J. Neurosci. 35, 1354–1367. 10.1523/JNEUROSCI.1418-14.2015 25632114 PMC4308588

[B75] Yoder R. M. Valerio S. Crego A. C. G. Clark B. J. Taube J. S. (2019). Bilateral postsubiculum lesions impair visual and nonvisual homing performance in rats. Behav. Neurosci. [Epub ahead of print]. 10.1037/bne0000321 31169384 PMC6721993

